# Patients' and Healthcare Personnel's Experiences of Health Coaching with Online Self-Management in the Renewing Health Project

**DOI:** 10.1155/2017/9306192

**Published:** 2017-12-31

**Authors:** Inger Lindberg, Birgitta Lindberg, Siv Söderberg

**Affiliations:** ^1^Division of Nursing, Department of Health Science, Luleå University of Technology, 971 87 Luleå, Sweden; ^2^Department of Nursing Sciences, Mid Sweden University, 83125 Östersund, Sweden

## Abstract

**Background:**

Telehealth applications have shown positive effects for people with chronic conditions and their awareness of health.

**Objective:**

To describe patients' and healthcare personnel's experiences of using health coaching with online self-management in primary health care.

**Method:**

A pragmatic randomised controlled trial was conducted. Patients in the intervention group measured and reported medical parameters such as blood pressure, blood glucose, prothrombin complex (PK) values, and 2-channel ECG. Data were collected through a questionnaire, individual interviews with patients, and focus group discussions with healthcare personnel. The questionnaire was analysed using statistics; texts from interviews and focus groups were analysed using content analysis.

**Findings:**

Patients were satisfied and believed that the intervention had enhanced their care and increased accessibility without causing concerns about privacy. Although being positive, patients commented the lack of support and feedback from healthcare personnel. Healthcare personnel regarded the intervention valuable for the patients' abilities to perform self-management healthcare tasks but preferred that patients did so without them supporting the patients.

**Conclusion:**

Patients expressed satisfaction and acceptance regarding the use of the application. It seems that healthcare personnel are convinced about the benefits for patients and the potential for the intervention but are not convinced about its benefits for healthcare organisations.

## 1. Introduction

The world prevalence of diabetes among adults (ages 20–79 years) is estimated to be 8.8 percent, affecting 415 million adults in 2015. This is expected to increase to 10.4 percent and 642 million people by 2040 [1]. It is estimated that more than 59.8 million people in the European region have diabetes; by 2040, this is expected to increase to 71.1 million. Of the 57 million global deaths in 2008, 36 million (63%) were due to noncommunicable diseases (NCDs) and 17.3 million (30%) were due to CVDs. Almost 80% of NCD deaths occur in low and middle income countries and it is the most frequent cause of death in most countries, except in Africa [2].

Careful glucose control, effective control of blood pressure, and effective management of risks in the presymptomatic phases of CVDs have been shown to reduce complications [3–6]. Prevention of complications of diabetes is about ensuring that patients have a good quality of life and includes lifestyle management such as diet and physical activity [7]. Controlling hypertension can decrease CV events such as coronary heart disease, congestive heart failure, stroke, and renal failure [3, 6].

Telehealth applications have shown positive effects for people with hypertension [8], CVD risks [9], and congestive heart failure [10, 11]. Furthermore, such applications influence patients' awareness about their own health [12, 13]. A review study found that technology intervention treatments result in positive behavioural changes among patients and are potentially highly beneficial for the management of chronic illnesses such as type 2 diabetes [14]. Another review supports the use of a self-management health information technology approach to improve glycaemic control. The effect of this approach was significantly greater when the technology used a web-based application, when a mechanism was provided for patients to enter their health data (manually or automatically), and when the technology was operated in the home or with no restrictions regarding location [15]. Furthermore, it was shown that telehealth was an effective model for the provision of diabetes care to rural patients compared to face-to-face visits [16]. Research on home health telemonitoring has also shown contradictory results; for example, Wakefield et al. [17] found that the addition of technology alone did not improve outcomes related to haemoglobin A1c.

When implementing technology in health care, there is a need for effective and responsive clinical processes to optimize the use of the additional data. In a study about implementing blood glucose and blood pressure in home health telemonitoring in primary care practices for patients with diabetes, stakeholder groups (patients, nurse care coordinators, and physicians) expressed that the careful consideration of workflow and information flow would help enable effective implementations [18]. It was also considered that practices need to understand the capabilities and limitations of the technology, the involvement of the patients and stakeholders, and their willingness to use it. In order to have successful implementation of different self-management applications, several perspectives must be considered. This study intends to add knowledge based on the views of both patients and healthcare personnel in regard to the online self-management of patients with chronic conditions. Thus, the aim of this study was to describe patients' and healthcare personnel's experiences of using health coaching with online self-management in primary health care.

## 2. Method

### 2.1. Context

The study was conducted in four healthcare centres in the county of Norrbotten in the northern part of Sweden. It comprises 25 percent of Sweden's land area and a population of 250,000 inhabitants. The study is a part of the Renewing Health project; the project included nine regions in Europe aiming to implement large-scale, real-life test beds for the validation and subsequent evaluation of innovative telemedicine services using a patient-centred approach and a common rigorous assessment methodology [19].

### 2.2. The Intervention

The Swedish field trial in the Renewing Health project developed a method that combined health coaching with the online management of patients' data, where the patient becomes more actively involved by self-monitoring his or her own health and health care via a national patient portal. The portal provides secure access to their health information for all Swedish citizens and supports electronic interaction with healthcare professionals. During the field trial, patients used their own computers; patients without access to a computer were provided with an iPad. The preventive health and diagnostic measurement equipment for self-monitoring were also provided to patients during the field trial.

The intervention was initiated with group sessions that aimed to educate and motivate patients to undertake lifestyle changes. The patients were trained to manage their health information and interact with the healthcare professionals through the application. A health activity plan was developed for each patient, and during the time frame established for the study, patients performed health-promoting activities and reported parameters such as the number of steps taken in a day, pulse, and the duration of physical activity. Based on their diagnoses, the patients measured and reported medical parameters such as blood pressure, blood glucose, prothrombin complex (PK) values, and 2-channel ECG. Approximately every second month, the results were reviewed and the health activity plan for each patient was revised if necessary. The patients then received feedback from the healthcare professional via either email or video. Feedback could be about reported medical parameters, changes in drug treatment, and physical activities. General practitioners, diabetes nurses, physiotherapists, and nutritionists cooperated to manage patient interactions.

### 2.3. Study Design

In this study, a pragmatic randomised controlled trial was conducted. The methodology used was in accordance with the model for assessment of telemedicine applications (MAST) [20]. This ensured that the outcomes with regard to safety, clinical impact, patient perception, economic aspects, and organisational aspects were assessed in accordance with scientific guidelines.

### 2.4. Participants and Procedure

#### 2.4.1. The Quantitative Study

In May 2011, 4796 eligible patients who met the inclusion criteria (Table 1) were contacted through an informational letter sent from their healthcare centres. Of these, 4057 were excluded due to their decisions to decline to participate or because they did not meet the inclusion criteria, leaving 739 participants. After the participants had signed an informed consent form, randomisation was performed following standard procedures (PC-based generation of random integer sequences and allocation based on consecutive assignment). Randomisation was performed by a statistician at the Research and Development Unit of the Norrbotten County Council. During the intervention period, 180 participants in the intervention group and 54 participants in the control group were lost to follow-up due to reasons that included being too ill, finding the technology too difficult to manage, changing healthcare centres, and death. At the end of the trial, 15 March 2013, all 253 participants in the intervention group were approached by the contact person at each primary healthcare centre, where they completed the questionnaire, which took about 15 minutes. Of the remaining 253 participants, 245 completed the questionnaire. The response rate was 96 percent.

#### 2.4.2. The Qualitative Study

A total of 33 patients (16 women and 17 men) from the four healthcare centres included in the Renewing Health project participated. The patients were between 45 and 89 years old (md = 66) and were diagnosed with either type 2 diabetes, heart disease, or hypertension. During spring of 2013, the contact person at each primary healthcare centre distributed an invitation letter to patients in the intervention group to participate in an interview study about their experiences of the intervention. Invitations were based on the participants having participated in or withdrawn from the study. Of 39 patients who were invited and 35 accepted, 2 declined later due to personal reasons. The researchers contacted the patients to schedule appointments to be interviewed at their convenience.

Four focus group discussions were held with the healthcare staff (*n* = 22) at the participating primary healthcare centres involved in the project. The participants were between 39 and 65 years old (md = 54) and had worked between 13 and 43 years (md = 24.5). The participants' professions included a biomedical scientist, diabetes nurses, district nurses/nurses, an occupational therapist, physicians, physiotherapists, a medical secretary, and enrolled nurses.

### 2.5. Data Collection

#### 2.5.1. Questionnaire, Service User Technology Acceptability Questionnaire (SUTAQ)

The questionnaire was developed by NHS England Whole System Demonstrator Programme [21] and was translated into Swedish and then back-translated to English. A pilot trial, which resulted in one change, was conducted with 10 people from an earlier e-health study. The questionnaire consists of 22 items (statements) in a Likert-type scale that includes both positive and negative statements in order to reduce the risk of bias. The topics include questions about the following: (1) enhanced care: did patients believe telemedicine enhanced the care they received? (2) Increased accessibility: did they think that it increased accessibility to healthcare services? (3) Privacy and discomfort: did they believe that it created problems related to their privacy and/or made them feel uncomfortable? (4) Care personnel concerns: did they have concerns about the personnel involved in the process of telemedicine? (5) Kit (here in this study; PC, iPad, blood pressure, pulse, step, and PK meters) as substitution: did they see it as a substitute to their usual care? (6) Satisfaction: were they satisfied with it overall? The answers to all items (questions) in the questionnaire were coded as follows: 1 = strongly agree, 2 = moderately agree, 3 = mildly agree, 4 = mildly disagree, 5 = moderately disagree, and 6 = strongly disagree.

#### 2.5.2. Interviews with the Patients

Personal interviews were conducted with 33 patients from the intervention group, both those who participated throughout the duration of the project (*n* = 27) and those who withdrew from participation (*n* = 6). The interviews were semistructured, and the participants were asked to talk about their experiences of communicating with the physician and/or diabetes nurse via a web application, their thoughts about getting care through computers or mobile phone, how they experienced the function of technology, and how they believed the application supported their self-care abilities. The interviews were digitally recorded; they lasted approximately 15 to 20 minutes and were later transcribed verbatim.

#### 2.5.3. Focus Group Discussions with Healthcare Personnel

The focus group discussions focused on how the healthcare staff experienced communicating with patients through the web application. The healthcare staff was asked to talk about their experiences of care through computers or mobile phones, how they experienced the technology, and whether or not the application supported patient self-care. The discussions were digitally recorded, lasted approximately 30 to 60 minutes, and were later transcribed verbatim.

### 2.6. Data Analysis

#### 2.6.1. Quantitative Method

*Questionnaire.* Based on the answers to the items, six subscales were identified as follows: enhanced care (based on items 17, 15, 10, 11, and 13); increased accessibility (based on items 1, 3, 4, and 19); privacy and discomfort scale (based on items 5, 2, 8, and 12); care personnel concerns (based on items 9, 21, and 20); kit as substitution (based on items 18, 22, and 16); and satisfaction (based on items 7, 6, and 14). The calculation of the scales was performed by subtracting the mean value of the items from 7.

For all subscales, the interpretation is that a higher value reflects a higher degree of agreement with the subscale. A high value on the subscale “satisfaction,” for example, 5.5, reflects a high degree of agreement and, therefore, a high degree of satisfaction. The two subscales “privacy and discomfort” and “care personnel concerns” are based on negative statements in the items. Therefore, a high value on these subscales reflects a high degree of agreement with this negative aspect of the kit. For example, a high value of, for example, 5.5, on the subscale “care personnel concerns” reflects that the patients are, to a high degree, concerned with regard to the personnel taking care of them by using the kit.

#### 2.6.2. Qualitative Method

*Interviews and Focus Group Discussions.* Both the interviews with the patients and the focus group discussions with the healthcare personnel were analysed with a qualitative content analysis [22]. The method can be described as a process of identifying, coding, and categorising the primary pattern of the data (i.e., the content). The data analysis started with reading each interview several times to get a sense of the content. After that, guided by the aim of the study, a reading followed to identify textual units. The textual units were then condensed and sorted into categories based on similarities in content. The categories were then related to each other and subsumed into final categories.

## 3. Ethical Approval

Informed consent was collected after the participants were informed orally and in writing about the study's purposes. Participants were assured that they could withdraw from the study without giving any explanation; that the results would not be linked to individuals; and that all study results would maintain confidentiality. The study was conducted according to the Ethical Review Act (The Ministry of Education and Cultural Affairs, 2003:460) and approved by the Regional Ethical Review Board (Dnr 2010/386-31M).

## 4. Results

The results are presented in two parts. In the first part, results from the questionnaire are presented (Table 2), and in the second part, the results from the interviews with the patients and the focus group discussions with the healthcare personnel are presented.

### 4.1. Part 1: Results of the Quantitative Analysis

In the subscale “enhanced care,” participants moderately agreed that the intervention would enhance their ability to take care of their own health or would be a good addition to their regular health care. The kit allowed them to be less concerned about their health, and they would recommend it to people with similar conditions. They also mildly to moderately agreed that the intervention increased accessibility to care and healthcare professionals. By using the kit, the participants saved time because they did not have to visit the GP clinic as often as before. The kit was perceived to increase access to care and made it easier to improve their health. Participants strongly disagreed about being concerned about their privacy and worrying about their private information being compromised by using the kit, and they expressed that they were comfortable using it. They did not perceive that the kit interfered with their everyday routines. They also moderately disagreed about being concerned about staff being competent enough or risking lack of continuity of care. They were not concerned that the healthcare personnel had access to their healthcare history. Participants mildly agreed that the kit was a positive addition to their regular health care and allowed them to be less concerned about their health status. They had concerns about the kit being suitable as a replacement for regular to face-to-face consultations. Overall, the participants strongly agreed that they were satisfied with the service with regard to the kit and that it was easy to understand and reliable.

### 4.2. Part II: Results of the Qualitative Analysis with Patients and Healthcare Personnel

#### 4.2.1. The Patients

The analysis of the interviews with the patients resulted in four categories as follows: experiencing the technology, the importance of feedback, self-care, and privacy. The results are illustrated with quotations from the interviews.

*Experiencing the Technology.* Participants described that using the technology was, overall, a positive experience and that it was both exciting and interesting. They considered the technology to be user friendly, and the general opinion was that it worked well. Several participants described that the technology was a bit difficult the first time they used it. One participant expressed, “The technology works well and the equipment works well and it was easy to use.” This was also described by patients with limited experience using computers. One participant said, “What surprised me was that there were many elderly people not skilled with computers, but it works well for them too.” Some participants even said that it had made them more interested in wanting to learn more about technology. 

*The Importance of Feedback.* Participants highlighted the importance of receiving feedback from healthcare staff when they had reported data. One participant said, “I don't believe that technology can be good if you do not have individual follow-up or feedback, but I think that the feedback can be handled by phone or mail; it's not necessary to have personal meetings.” The participants described varying experiences regarding how the feedback had worked, but many said they wished that the feedback had been better. One participant expressed, “No feedback at all. You could see if someone [the healthcare staff] had been checking it, but there was never anyone who had been checking it. It felt like sending [data] into a black hole.” There were also participants who had the opposite opinion, as one participant said, “I always got feedback on reported measurements, so they [the healthcare staff] had control.”

*Self-Care.* Participants expressed varying opinions regarding self-care; some patients said that their health had improved, and others said that there was no difference. One participant said, “My health has definitely improved because I understand the importance of exercise.” Another said, “I don't feel that my health has improved.” The participants agreed that using the technology gave them increased control and insight about the importance of how exercise affects their health. They stated that reporting their physical activities motivated them to exercise more often than before. One participant expressed, “It engages yourself in a different way and makes you feel confident when you have control. This is two very positive things.” The participants also stated that having control of their own health and measurements gave them a sense of security or safety. The participants expressed that health care provided this way will be part of the future and that it will expand. One participant expressed, “In the future, it will become even more of this. Yes, especially for those who live at some distance from the healthcare centre.”

*Privacy.* The participants were consistent in regard to not being worried about their privacy being threatened. One participant said, “No. no, no, I don't feel that my privacy has been threatened. If there is anyone who wants to read, let them.” Another said, “I've never even had a thought about it being an intrusive invasion of privacy.” They also expressed that the reported measurements were not a secret or something they needed to hide from others. As one participant said, “It's not a secret; there is nothing to be ashamed of.”

*Experiences of People Who Withdrew from Participation.* The interviews with those who withdrew from participation in the intervention showed similar experiences to those who participated throughout the duration of the project. Some reasons for withdrawing are shown in the quotations. As one participant said, “I changed healthcare centres so I could not continue my participation. I wanted to continue.” Another participant expressed, “I had problems with my e-ID so I chose to leave, but I think it was due to my own lack of knowledge.”

#### 4.2.2. The Healthcare Personnel

The analysis of the focus group discussions with the healthcare personnel resulted in five categories as follows: reluctance to change the way they have been working, strengthening self-care, opinions about the application, and something for the future. The categories are presented below supported by quotations from the discussions.

*Reluctance to Change the Way They Have Been Working.* Healthcare personnel emphasized that they were forced to change their working methods and found it difficult to work in this new way. One participant said, “One has always a certain resistance to do something new.” Another participant said, “It's a tradition hard to change…you're used that the patient visits the health centre.” Healthcare staff described that they used the application when the patient visited the healthcare centre, which means that the staff used the application in the conventional care mode. They did not follow up on the data the patient registered in the application that was available on the national patient portal. As one participants expressed, “…have commented on it occasionally,… I've used it when I met a patient and used it as their own control.”

*Strengthening Self-Care.* Strengthening self-care was something that the healthcare personnel thought of as beneficial for the patients. One participant said, “Now the patients can use the system … and it's for their own benefit and care.” The healthcare personnel expressed that the advantage with the application was that it made the patient more aware of his or her own health and self-care. It was a possibility for the patients to notice when they exercised and to follow their blood pressure or their blood sugar. As one participant stated, “It's for them.”

*Opinions about the Application.* The healthcare personnel had many opinions about the application. They expressed that in the beginning it was difficult to use because it did not function properly all the time. The healthcare personnel considered that there were too many steps they had to take before they could enter and start to work with the application. Some of the healthcare personnel expressed that to use the application in their daily work was a generational issue. As one participant said, “I think it's a generational issue. Should I be able to use it, it must be very easy to follow. Basically self-explanatory.”

*Something for the Future.* The healthcare personnel highlighted that this method of working was something for the future. They believed that in several years the patients would be ready to master the technology. The healthcare personnel said, “you just have to look at your grandchildren and how they handle the computer and iPad and whatever it is called.” They also expressed that support from the physicians was essential for the implementation of this method of working. Several participants expressed the importance of support from doctors.

## 5. Discussion

The main finding in the study is that both healthcare personnel and patients experienced the intervention to be valuable and to function well, but it is evident that there were different expectations about the fulfilment of the intervention. Patients expected feedback and support, while healthcare staff expected patients to perform self-management tasks mostly without staff support.

Thus, healthcare personnel regarded the intervention as valuable for patients to perform self-management but preferably without them supporting the patients. They meant that the intervention enabled them to save time and focus on more severely ill patients. According to a review study, relatively little research attention has been given to the effects of e-health on roles and responsibilities, risk management, ways to engage with professionals, and ensuring that the potential benefits of new technologies are made transparent through the interventions' evaluations and feedback [23]. Greater focus should be directed to empirical investigations in order to identify and anticipate how e-health services will impact everyday clinical practices and to examine how new e-health services will affect clinical interactions and activities and the allocation and performance of clinical work, as well as different methods of engaging with professionals before and during the implementation of e-health services [18, 23, 24]. The results of a study comparing the Agile Process and the Incremental Process indicate that development performance and product quality achieved by following the Agile Process were superior to those achieved by following the Incremental Process in the projects compared [25]. Agile models offer a smooth and flexible approach aiming to respond to changing requirements; adjustments to supportive tools under development can be adjusted during the process as needed. It is beneficial when requirements are instable and constantly changing. The Incremental model is a modified version of the Waterfall model, where the product is developed incrementally in parts as a response to increasing product sizes and are delivered in sequences, each with a wider functionality. It is beneficial when requirements are incomplete at the start of the project and elicited in time. [26].

Patients in the intervention group were highly satisfied, believing that the intervention enhanced their care and increased accessibility without causing concerns about privacy, discomfort, or the involvement of personnel. In general, however, it was not seen as a substitute for their usual care, although it was regarded as comparable to an “in room meeting.” This is in line with the results of a literature review [27] showing that patients prefer a combination of telecare and traditional healthcare delivery. Therefore, telemedicine must be used as a complement and not a replacement for standard health care. According to Bardram et al. [28], information and communication technology (ICT) used in home health care must take into consideration the role technology should play in its use by patients and healthcare professionals. Home monitoring devices or systems can help support patients to have greater flexibility in their lives and to be more independent when managing their medical conditions. If patients feel that home-based self-care benefits and empowers them, they are more likely to continue to use it [29].

Although generally positive about handling the technical application and performing self-management care, the patients in the study commented strongly that they lacked support and feedback about the registered data from the healthcare personnel, and they felt that this left them in limbo. For telehealth to be a successful tool, it is necessary to ensure adequate follow-up and feedback to the patient [29, 30]. Without adequate participant education or with low health-literacy levels, the result tends to be lower compliance, while active participation with human support from a healthcare service provider reinforces good compliance behaviour [29]. The results in one study showed that elderly and/or chronically ill persons who have home telecare contacts at fixed times every day perceive greater advantages of home telecare; they also perceive the technology as more compatible and less complex, and they perceive more observability compared to clients who have home telecare contacts based only on their own initiative [31].

Healthcare personnel in the study thought that the intervention was something for the future and did not change their way of organising care work in order to support patients using the application. If they used the application, it was used in conventional care. According to Miller, healthcare personnel have to see the benefits of ICT; otherwise, they might have difficulty seeing the technology as part of their daily work routine [32]. Another study demonstrated that when barriers to successful implementation exist, healthcare personnel can lose their faith in using technology, for example, telemonitoring for patients with chronic illnesses, and instead perform tasks traditionally delivered face-to-face [33]. Addressing such barriers is of critical importance for the successful implementation of telehealth in routine healthcare practices. In an evaluation of a telehealth service for patients with chronic obstructive pulmonary disease and heart failure, several barriers were identified to affect the level of telehealth adoption by patients, including a preference for face-to-face physical contact with their healthcare personnel, technology anxiety, technical problems, and the belief that telehealth is unnecessary [34].

There were a considerable number of dropouts in the intervention group. Reasons included becoming too ill, finding the technology too difficult to handle, changing healthcare centres, and death. In a meta-analysis, the reasons for patients dropping out or withdrawing are important to recognise, as they can significantly inform the future development and design of information technology used for healthcare treatments [14]. Furthermore high amount of dropouts can compromise the validity of the results. Improvements in telemedicine require knowledge and understanding of how users (patients) physically and emotionally interact with and react to the technologies [18]. In our study, the information about the reasons for dropouts and withdrawals shows that more careful consideration is required before offering this kind of electronic healthcare service.

## 6. Conclusion

The main conclusion remains that telemedicine, for example, the use of ICT in healthcare services, is associated with high user satisfaction and acceptability by patients, a very significant result in an era of indisputable consumer sovereignty and patient-centred healthcare systems. It seems that healthcare personnel are convinced about ICT's benefits for patients and that this is something for the future. However, they are not convinced about its benefits for healthcare organisations. For the successful implementation of telemedicine, organisational changes are necessary.

## Figures and Tables

**Table 1:** Inclusion criteria.

Diabetes type 2	CVD	Hypertension
Diagnosed > three months prior to the enrolment	Diagnosis of ischemic heart disease	Diagnosis of hypertension
HbA1c > 6.5%		
Age > 18 years	Age > 18 years	Age > 18 years
Capability to fill in questionnaires in their own language	Capability to fill in questionnaires in their own language	Capability to fill in questionnaires in their own language
Capability to use the devices provided	Capability to use the devices provided	Capability to use the devices provided
Being cognitively able to participate	Being cognitively able to participate	Being cognitively able to participate

**Table 2:** Results questionnaire, Service User Technology Acceptability Questionnaire (SUTAQ).

Subscales	Diabetes *n* = 51	CVD *n* = 48	Hypertension *n* = 154
*Enhanced care*	5.10 (0,63), 5.2 *n* = 48	4.78 (1,01), 4.8 *n* = 45	5.04 (0,85), 5.2 *n* = 149
Missing	3	3	5
*Increased accessibility*	4.55 (1,07), 4.8 *n* = 48	4.15 (1,34), 4.5 *n* = 45	4.49 (1,10), 4.8 *n* = 149
Missing	3	3	5
*Privacy and discomfort*	1.53 (0,66), 1.3 *n* = 48	1.43 (0,51), 1.3 *n* = 46	1.51 (0,60), 1.3 *n* = 151
Missing	3	2	3
*Care personnel concerns*	1.97 (0,85), 2.0 *n* = 48	1.93 (1,01), 1.7 *n* = 44	2.01 (0,99), 1.7 *n* = 151
Missing	3	4	3
*Kit as substitution*	3.96 (0,83), 4.0 *n* = 47	3.45 (1,01), 3.7 *n* = 45	3.82 (0,88), 4.0 *n* = 150
Missing	4	3	4
*Satisfaction*	5.68 (0,52), 6.0 *n* = 47	5.41 (0,94), 5.7 *n* = 46	5.57 (0,70), 6.0 *n* = 149
Missing	4	2	5
